# Supplementary material for: Combined lineage tracing and scRNA-seq reveals unexpected first heart field predominance of human iPSC differentiation
Source: eLife. 2023 Jun 7;12:e80075. doi: 10.7554/eLife.80075 (PMC10348749; doi:10.7554/eLife.80075)
Supplement: MDAR checklist [file elife-80075-mdarchecklist1.docx]

**Materials Design Analysis Reporting (MDAR)**

**Checklist for Authors**

The [MDAR framework](https://osf.io/xfpn4/) establishes a minimum set of requirements in transparent reporting mainly applicable to studies in the life sciences.

*eLife* asks authors to **provide detailed information within their article** to facilitate the interpretation and replication of their work. Authors can also upload supporting materials to comply with relevant reporting guidelines for health-related research (see [EQUATOR Network](http://www.equator-network.org/%20)), life science research (see the [BioSharing Information Resource](http://biosharing.org/)), or animal research (see the [ARRIVE Guidelines](http://www.plosbiology.org/article/info:doi/10.1371/journal.pbio.1000412) and the [STRANGE Framework](https://doi.org/10.1038/d41586-020-01751-5); for details, see *eLife*’s [Journal Policies](https://reviewer.elifesciences.org/author-guide/journal-policies)). Where applicable, authors should refer to any relevant reporting standards materials in this form.

For all that apply, please note **where in the article** the information is provided. Please note that we also collect information about data availability and ethics in the submission form.

**Materials:**

| **Newly created materials** | **Indicate where provided: section/figure legend** | **N/A** |
| --- | --- | --- |
| The manuscript includes a dedicated "materials availability statement" providing transparent disclosure about availability of newly created materials including details on how materials can be accessed and describing any restrictions on access. | We provide a dedicated “Materials and Methods” section where we include a statement of unrestricted access to all plasmids constructed for the study. All materials are freely available to scientific community upon request. Figure 1 provides a detailed description of the genetic donor constructs engineered for our study. We also provide supplementary file 10 which contains all single guide RNA sequences used for genome editing. |  |
|  |  |  |
| **Antibodies** | **Indicate where provided: section/figure legend** | **N/A** |
| For commercial reagents, provide supplier name, catalogue number and [RRID](https://scicrunch.org/resources), if available. | Under “Materials and Methods” section, under “Immunofluorescence Staining” and “Flow Cytometry” catalogue numbers and suppliers have been listed for all antibodies used in staining protocols. |  |
|  |  |  |
| **DNA and RNA sequences** | **Indicate where provided: section/figure legend** | **N/A** |
| Short novel DNA or RNA including primers, probes: Sequences should be included or deposited in a public repository. | RT-qPCR primer sequences used in this study are provided under Supplementary File 11. We provide sequences for Hashtag oligos used for single cell RNA sequencing multiplexing under Supplementary File 12. We provide sequences of genetic construct primer inside-outside PCR in Supplementary File 16. |  |
|  |  |  |
| **Cell materials** | **Indicate where provided: section/figure legend** | **N/A** |
| Cell lines: Provide species information, strain. Provide accession number in repository OR supplier name, catalog number, clone number, OR RRID. | Human induced pluripotent stem cell lines used in this study were obtained from the Stanford Cardiovascular Institute Biobank (SCVI-111, Sendai virus reprogrammed peripheral blood mononuclear cells, healthy male with normal karyotype, 46, XY). The WTC-11 (reprogrammed from healthy males with normal karyotype, 46, XY) hiPSC line was provided by Bruce Conklin’s laboratory at the University of California, San Francisco and has been deposited into the Coriell Institute for Medical Research under identifier GM25256. For SCVI-111, G-banding karyotyping was conducted and cell line identity was confirmed by short tandem repeat analysis of the cell line and donor PBMCs. For the WTC-11 line, G-banding karyotyping was conducted and cell line identity was confirmed by short tandem repeat analysis of the cell line to donor fibroblasts. All cell lines tested negative for mycoplasma. Studies involved human iPSCs approved under protocol #460 of the Stanford Stem Cell Research Oversight (SCRO) committee. Please see “Cell Lines” under the “Materials and Methods” section. |  |
| Primary cultures: Provide species, strain, sex of origin, genetic modification status. | Not applicable | N/A |
|  |  |  |
| **Experimental animals** | **Indicate where provided: section/figure legend** | **N/A** |
| Laboratory animals or Model organisms: Provide species, strain, sex, age, genetic modification status. Provide accession number in repository OR supplier name, catalog number, clone number, OR RRID. | Not applicable | N/A |
| Animal observed in or captured from the field: Provide species, sex, and age where possible. | Not applicable | N/A |
|  |  |  |
| **Plants and microbes** | **Indicate where provided: section/figure legend** | **N/A** |
| Plants: provide species and strain, ecotype and cultivar where relevant, unique accession number if available, and source (including location for collected wild specimens). | Not applicable | N/A |
| Microbes: provide species and strain, unique accession number if available, and source. | Not applicable | N/A |
|  |  |  |
| **Human research participants** | **Indicate where provided: section/figure legend) or state if these demographics were not collected** | **N/A** |
| If collected and within the bounds of privacy constraints report on age, sex, gender and ethnicity for all study participants. | No human research participants were included in this study. |  |

**Design:**

| **Study protocol** | **Indicate where provided: section/figure legend** | **N/A** |
| --- | --- | --- |
| If the study protocol has been pre-registered, provide DOI. For clinical trials, provide the trial registration number OR cite DOI. | Not applicable | N/A |
|  |  |  |
| **Laboratory protocol** | **Indicate where provided: section/figure legend** | **N/A** |
| Provide DOI OR other citation details if detailed step-by-step protocols are available. | For making of in-house E8 media we provide citation to previously published protocol: <https://doi.org/10.1002/0471142905.hg2103s87>  For genome editing protocol used in our study we provide citation to step-by-step protocol: <https://doi.org/10.1007/978-1-0716-0668-1_17> |  |
|  |  |  |
| **Experimental study design (statistics details) *** | | |
| **For in vivo studies: State whether and how the following have been done** | **Indicate where provided: section/figure legend. If it could have been done, but was not, write “not done”** | **N/A** |
| Sample size determination | No in vivo studies were conducted |  |
| Randomisation | No in vivo studies were conducted |  |
| Blinding | No in vivo studies were conducted |  |
| Inclusion/exclusion criteria | No in vivo studies were conducted |  |
|  |  |  |
| **Sample definition and in-laboratory replication** | **Indicate where provided: section/figure legend** | **N/A** |
| State number of times the experiment was replicated in the laboratory. | We provide clear statements of the number of independent biological replicates that were conducted for each experiment. In figure legends for Figures 2 and 3 we state the number of biological replicates and present each individual replicate on their respective bar graphs. Minimum of 3-4 biological replicates were collected per timepoint or cell line evaluated. |  |
| Define whether data describe technical or biological replicates. | We indicate in figures 2 and 3 legends the number of biological replicates that were collected per cell line and timepoint. In the “Materials and Methods” section under the “Statistics” subsection we describe howe have defined biological and technical replicates in our study. |  |
|  |  |  |
| **Ethics** | **Indicate where provided: section/submission form** | **N/A** |
| Studies involving human participants: State details of authority granting ethics approval (IRB or equivalent committee(s), provide reference number for approval. | Approval for studies using human induced pluripotent stem cells were approved under protocol #460 of the Stanford Stem Cell Research Oversight Committee as described in “Materials and Methods” section under “Cell Lines”. |  |
| Studies involving experimental animals: State details of authority granting ethics approval (IRB or equivalent committee(s), provide reference number for approval. | Not applicable | N/A |
| Studies involving specimen and field samples: State if relevant permits obtained, provide details of authority approving study; if none were required, explain why. | Not applicable | N/A |
|  |  |  |
| **Dual Use Research of Concern (DURC)** | **Indicate where provided: section/submission form** | **N/A** |
| If study is subject to dual use research of concern regulations, state the authority granting approval and reference number for the regulatory approval. | Not applicable | N/A |

**Analysis:**

| **Attrition** | **Indicate where provided: section/figure legend** | **N/A** |
| --- | --- | --- |
| Describe whether exclusion criteria were pre-established. Report if sample or data points were omitted from analysis. If yes, report if this was due to attrition or intentional exclusion and provide justification. | Outliers were excluded during analysis of flow cytometry data to exclude cardiac differentiations that deviated significantly from distribution observed. To eliminate outliers we used the ROUT method as described in “Materials and Methods” under “Statistics.” This method was used in order achieve unbiased outlier detection. |  |
|  |  |  |
| **Statistics** | **Indicate where provided: section/figure legend** | **N/A** |
| Describe statistical tests used and justify choice of tests. | Statistical tests used for analysis in manuscript are described in the “Statistics” section under “Materials and Methods”. |  |
|  |  |  |
| **Data availability** | **Indicate where provided: section/submission form** | **N/A** |
| For newly created and reused datasets, the manuscript includes a data availability statement that provides details for access (or notes restrictions on access). | We provide GEO accession numbers for the datasets generated in this manuscript under the “Data Availability Statement” under “Materials and Methods” |  |
| When newly created datasets are publicly available, provide accession number in repository OR DOI and licensing details where available. | The GEO accession is as follows: GSE202398 |  |
| If reused data is publicly available provide accession number in repository OR DOI, OR URL, OR citation. | For publicly available data used in our study we provide the accession number under Supplementary File 9. |  |
|  |  |  |
| **Code availability** | **Indicate where provided: section/figure legend** | **N/A** |
| For any computer code/software/mathematical algorithms essential for replicating the main findings of the study, whether newly generated or re-used, the manuscript includes a data availability statement that provides details for access or notes restrictions. | We provide a “Data and Code Availability Statement” where we indicate the Github repository link to standard code used for analysis of single cell RNA-seq data in this manuscript. Please see “Materials and Methods” section. |  |
| Where newly generated code is publicly available, provide accession number in repository, OR DOI OR URL and licensing details where available. State any restrictions on code availability or accessibility. | No new code was generated. We provide links for functions and pipelines provided by developers of packages used in this study. Under “Data and Code Availability” section. |  |
| If reused code is publicly available provide accession number in repository OR DOI OR URL, OR citation. | All previously published packages used for the analysis of data in this manuscript have been appropriately cited throughout the manuscript. Detailed description of the version numbers and pipelines used for data analysis using publicly available packages are described in “Materials and Methods” section of manuscript. |  |

**Reporting:**

The MDAR framework recommends adoption of discipline-specific guidelines, established and endorsed through community initiatives.

| **Adherence to community standards** | **Indicate where provided: section/figure legend** | **N/A** |
| --- | --- | --- |
| State if relevant guidelines (e.g., ICMJE, MIBBI, ARRIVE, STRANGE) have been followed, and whether a checklist (e.g., CONSORT, PRISMA, ARRIVE) is provided with the manuscript. |  |  |

* We provide the following guidance regarding transparent reporting and statistics; we also refer authors to [Ten common statistical mistakes to watch out for when writing or reviewing a manuscript](https://doi.org/10.7554/eLife.48175).

**Sample-size estimation**

- You should state whether an appropriate sample size was computed when the study was being designed
- You should state the statistical method of sample size computation and any required assumptions
- If no explicit power analysis was used, you should describe how you decided what sample (replicate) size (number) to use

**Replicates**

- You should report how often each experiment was performed
- You should include a definition of biological versus technical replication
- The data obtained should be provided and sufficient information should be provided to indicate the number of independent biological and/or technical replicates
- If you encountered any outliers, you should describe how these were handled
- Criteria for exclusion/inclusion of data should be clearly stated
- High-throughput sequence data should be uploaded before submission, with a private link for reviewers provided (these are available from both GEO and ArrayExpress)

**Statistical reporting**

- Statistical analysis methods should be described and justified
- Raw data should be presented in figures whenever informative to do so (typically when N per group is less than 10)
- For each experiment, you should identify the statistical tests used, exact values of N, definitions of center, methods of multiple test correction, and dispersion and precision measures (e.g., mean, median, SD, SEM, confidence intervals; and, for the major substantive results, a measure of effect size (e.g., Pearson's r, Cohen's d)
- Report exact p-values wherever possible alongside the summary statistics and 95% confidence intervals. These should be reported for all key questions and not only when the p-value is less than 0.05.

**Group allocation**

- Indicate how samples were allocated into experimental groups (in the case of clinical studies, please specify allocation to treatment method); if randomization was used, please also state if restricted randomization was applied
- Indicate if masking was used during group allocation, data collection and/or data analysis
